# Fear-specific leftward bias in gaze direction judgment

**DOI:** 10.1038/s41598-021-97039-3

**Published:** 2021-09-02

**Authors:** Yue Zhang, Qiqi Hu, Xinwei Lai, Zhonghua Hu, Shan Gao

**Affiliations:** 1 Institute of Brain and Psychological Sciences, Sichuan Normal University, Chengdu, 610068 People’s Republic of China; 2 Research Center of Brain and Cognitive Neuroscience, Liaoning Normal University, Dalian, People’s Republic of China; 3 School of Foreign Languages, University of Electronic Science and Technology of China, Chengdu, 611731 People’s Republic of China; 4 The Clinical Hospital of Chengdu Brain Science Institute, MOE Key Laboratory for NeuroInformation, University of Electronic Science and Technology of China, Chengdu, People’s Republic of China

**Keywords:** Neuroscience, Psychology

## Abstract

Previous studies have shown that humans have a left spatial attention bias in cognition and behaviour. However, whether there exists a leftward perception bias of gaze direction has not been investigated. To address this gap, we conducted three behavioural experiments using a forced-choice gaze direction judgment task. The point of subjective equality (PSE) was employed to measure whether there was a leftward perception bias of gaze direction, and if there was, whether this bias was modulated by face emotion. The results of experiment 1 showed that the PSE of fearful faces was significantly positive as compared to zero and this effect was not found in angry, happy, and neutral faces, indicating that participants were more likely to judge the gaze direction of fearful faces as directed to their left-side space, namely a leftward perception bias. With the response keys counterbalanced between participants, experiment 2a replicated the findings in experiment 1. To further investigate whether the gaze direction perception variation was contributed by emotional or low-level features of faces, experiment 2b and 3 used inverted faces and inverted eyes, respectively. The results revealed similar leftward perception biases of gaze direction in all types of faces, indicating that gaze direction perception was biased by emotional information in faces rather than low-level facial features. Overall, our study demonstrates that there a fear-specific leftward perception bias in processing gaze direction. These findings shed new light on the cerebral lateralization in humans.

## Introduction

The spatial biases in human cognition and behavior are regarded as adaptive features which have gradually evolved in time^1^. While in the early stages of evolution, the biases are likely to reflect an early division in primary survival functions of the left and right hemispheres of the brain, in modern human development, the features may mainly indicate the cerebral lateralization in social functions. For example, 66–72% of human beings show a pronounced tendency to cradle babies on the left side of their own bodies, irrespective of handedness and sex^2,3^. Most individuals have a preference to turn heads to the right side of themselves when kissing^4^. When extracting social information from others’ faces, like gender^5^ and expression^6^, observers make more use of clues from their left-sided space (the right half part of the observed face).

In social cognition, the eyes provide vital information of others and gaze direction is one of the fundamental sources for social communication^7^. As an important spatial attention allocation cue and social signal, others’ gaze direction reflects their focus of interest and state of mind and forecasts their follow-up behaviors^8,9^. Thus, accurate judgment of others’ gaze direction is a critical social skill to facilitate the inference of the individuals^10^.

In practice, however, accurate judgment of gaze direction is not always achieved. Instead, humans have a bias in processing gaze direction^8^. Previous studies have revealed that humans tend to have a false belief of being directly looked at when the gaze direction is in fact slightly diverted towards left or right. Furthermore, and this bias interacts with face emotion in both adults^11–14^ and children ^15^. On the other hand, visual field has been shown to influence gaze direction processing. Ricciardelli et al.^16^ asked normal adults to make judgments for seen gaze direction (left, right or straight), with eye stimuli presented unilaterally (left or right eye) or bilaterally (i.e., two eyes gazed in the same or different directions). In the unilateral trials, performance was more accurate for stimuli in the left visual field (LVF) than those in the right visual field (RVF). With two eyes gazing in different directions, the LVF eyes affected the judgments more than the RVF eyes. These results suggested that the LVF has a greater impact on perception of gaze direction than the RVF. Greene and Zaidel^17^ and Marotta et al*.*^18^ found that the gaze cueing effect was significant when the gaze cue and target were presented in the LVF, but not in the RVF.

Pseudoneglect depicts the tendency to exhibit a subtle bias of visual attention favoring left space and such a leftward attentional bias is approved by various studies^19,20^. There is evidence showing that when processing nonsocial stimuli (i.e., line), subjects were influenced by pseudoneglect, leading to slightly leftward judgment when estimating the center of a line^20^. This left space preference may be due to the right hemisphere dominance in spatial allocation of attention^21^. Evidence from neuroimaging studies has suggested that the right hemisphere also plays a dominant role in processing of gaze direction given that brain regions in the right hemisphere including the right posterior superior temporal sulcus and right fusiform gyrus^22^, right amygdala^23^, and right inferior frontal junction^24^ have been observed to be more activated in gaze direction processing than those in the left hemisphere. Considering the dominance of the right hemisphere in both spatial allocation of attention and perception of gaze direction, we investigated whether there would be a leftward bias in perception of gaze direction, namely whether faces would be judged as looking at observers’ left-side space.

Based on previous animal studies, the right hemisphere is dominant in response to novel and threatening information in the environment^25–27^. Evidence from human research has shown a similar hemispheric lateralization in emotion processing, that is, the right hemisphere underpins negative emotion processing while the left hemisphere supports positive emotion processing^28^. Armaghani et al*.*^29^ asked participants to bisect horizontal lines, at both ends of which presented every combination of sad, happy, and neutral faces that participants were never required to attend to. The results showed that presentation of emotional faces as compared to neutral ones deviated line bisections more to the left of actual midline and the leftward deviation was greater with sad faces than with happy faces. Using the same task, on the other hand, Cattaneo et al*.*^30^ revealed that prolonged exposure to happy stimuli shifted the bisection bias to the right compared with both sad and neutral stimuli while there were no significant differences between sad and neutral stimuli. Given the valence-dependent hemispheric specialization, the present study included an emotional variable to investigate whether the magnitude of the leftward bias we expected to occur in perception of gaze direction would be modulated by face emotion.

We employed a forced-choice judgment task where participants were asked to judge whether virtual faces were looking left or right. Here, ‘left’ or ‘right’ was defined as the left-sided or right-sided space of observers/participants, not the left-sided or right-sided space of facial stimuli. There were ten different gaze directions and four different (angry, fearful, neutral, and happy) expressions of faces. Considering emotion-induced variations in both perception of gaze direction^12,14,15,31^ and spatial allocation of attention^29,30^, we first predicted that the magnitude of leftward perception bias in gaze direction judgment (i.e., judging faces as gazing at observers’ left side) would be larger for faces with negative expressions than for those with positive and neutral expressions. We also hypothesized differences between fearful and angry faces since they express two distinct negative emotions and associate with different motivations. Fear is an avoidance-oriented emotion, and in contrast, anger is an approach-oriented emotion^32^, leading to differential alterations of gaze direction perception^12^. Compared with angry faces, fearful ones are less likely to be judged as having direct gaze^14,31^. Therefore, we expected a larger leftward perception bias for fearful faces.

## Experiment 1

### Methods

#### Participants

Forty-two participants (23 females, *M* ± SD = 20.3 ± 5.6 years) were recruited from Liaoning Normal University of China. We determined the sample size by referring to previous studies^33–38^, in which 35–58 participants were included. Experiment 1 primarily aimed to obtain 40 participants. Considering the possibility of data exclusion, we tested two more participants during data collection. Therefore, the final sample size of experiment 1 was 42. Participants had normal or corrected-to-normal vision and were all right-handed according to their self-report. Signed informed consent was provided by each participant before the experiment. The study was conducted in accordance with the Declaration of Helsinki and was approved by the ethics committee of Liaoning Normal University (the approval number: lNNUIRB1713).

#### Materials

**Figure 1:**
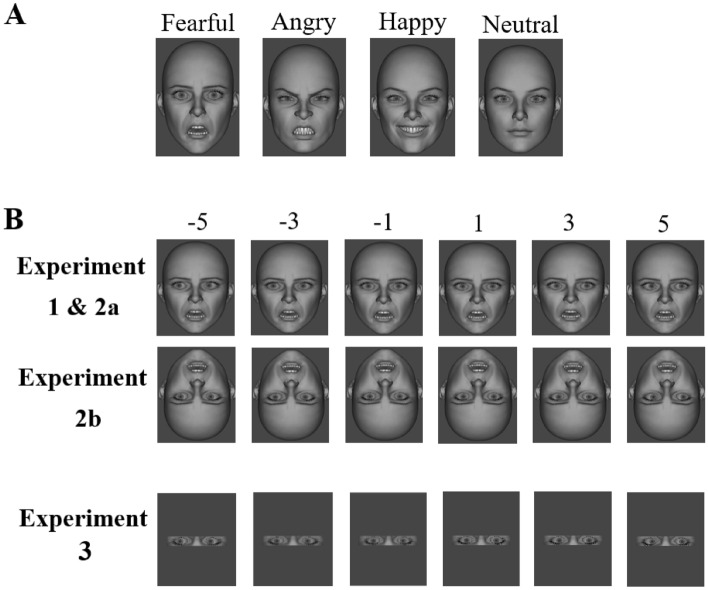
Facial images used as materials in experiments 1, 2a, 2b, and 3. (**A**) Examples of facial expressions with direct gaze. (**B**) Examples of gaze directions in fearful faces. Six of ten gaze directions are depicted (1, 3, 5 units to the left and 1, 3, 5 units to the right) with negative values indicating left averted gaze and positive values indicating right averted gaze. The upper part of panel B represents face stimuli used in experiments 1 and 2a, the middle part depicts examples of inverted faces used in experiment 2b and the lower part indicates examples of inverted eyes used in experiment 3.

Virtual face images were produced by DAZ Studio (https://www.daz3d.com/). We used *Genesis 2 Base Female* model and *Genesis 2 Base Male* model in DAZ Studio to create six identity faces (3 male faces and 3 female faces) with 4 facial expression (angry, fearful, happy, neutral) and 10 gaze directions (left 5, 4, 3, 2, 1 units; right 5, 4, 3, 2, 1 units) (see Fig. 1). A unit means a step. The distance between the center of the eye and the corner of the eye of the virtual face is divided into 10 parts. The center of the eye is 0 and the corner of the eye is 10. Right/left 1 unit means that the iris moves 1 step to the right/left. All facial images against a grey background were resized to 245 pixels in width and 344 pixels in height and subtended a visual angle of 8.43° by 10.03° on screen.

All the facial images were pretested by 13 volunteers (8 females) in terms of emotional category, valence, and arousal. The procedure of the face evaluation task was the same as described in Hu et al.^39^. The results of emotional category evaluation showed that the accuracy for the four emotional types of facial images was all above 90% and displayed no significant difference, *F*(1.482,17.784) = 1.005, *p* = 0.36. Rated on 9-point scales, happy faces had the highest emotional valence, angry faces the lowest, and fearful faces were evaluated lower than neutral ones. The valence differences were significant between any two emotions, *p*s < 0.05. In terms of arousal, neutral faces were rated significantly lower than all the other three emotions, *p*s < 0.01 while no significant differences were found between fearful, angry, and happy faces, *p*s > 0.05. The details of all the evaluation results are shown in Table 1.

**Table 1:** Results of pilot stimuli evaluation.

Type of face (M ± SD)					*p*	${\eta }_{p}^{2}$
	Fearful	Angry	Happy	Neutral		
Accuracy (%)	97 ± 0.04	92 ± 0.17	100 ± 0.01	97 ± 0.09	0.36	0.08
Valence	2.1 ± 0.52	1.9 ± 0.77	7.3 ± 1.13	4.9 ± 0.20	< 0.001	0.92
Arousal	6.6 ± 1.02	6.7 ± 0.78	6.4 ± 1.01	2.6 ± 1.25	< 0.001	0.81

Accuracy means the percentage in which participants correctly judged the type of emotion.

#### Procedure

The stimuli were presented with E-prime 2.0 Professional (https://pstnet.com/products/e-prime-legacy-versions/) on a 19-inch LCD monitor (1024 × 768 pixel, 60 Hz refresh rate). Participants seated 57 cm from the monitor and their heads were stabilized on a chin rest so that their eyes could be level with the eyes of stimuli faces. Free movements were prohibited.

At the beginning of each trial, the central fixation point “+” (1° × 1°) was presented and lasted for 1000 ms. Then, a stimulus face followed for 500 ms. Next, a blank grey screen was continuously displayed until participants’ response. Participants were instructed to judge whether the face was looking to their left or right side. If left, participants pressed the number key 1 using the right index finger; if right, participants pressed 2 using the right middle finger. Participants started with a practice session of 10 trials. The experimental session included six blocks of 120 trials, which were presented in a randomized order.

#### Data analysis

**Figure 2:**
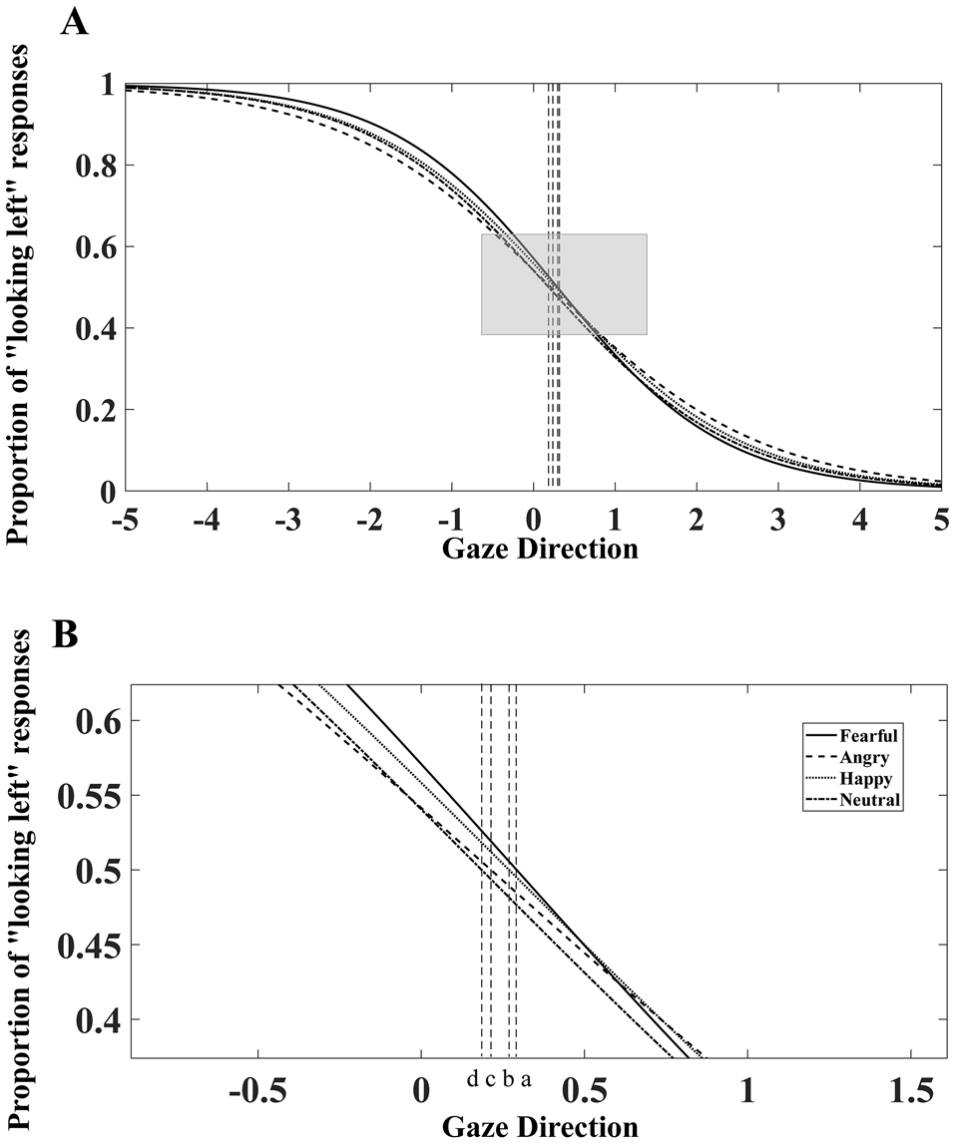
Grand mean plot (averaged across three experiments) showing the measurement of the point of subjective equivalence (PSE). (**A**) Depicts the fitted curves for “looking left” responses. (**B**) Is a magnified view of the shaded part of the fitted performance curves. The cross points *a, b, c,* and *d* between the vertical dashed lines and the x-axis represent PSE values in fearful, happy, angry, and neutral conditions.

The psychometric (logistic) functions were fitted to the proportion of “looking left” responses for each type of emotional faces with 10 gaze directions (see supplementary material), and the coefficients of a nonlinear regression function were estimated using least squares estimation. The points of subjective equivalence (PSE) were calculated as the point of gaze direction which an individual judged as “looking left” with 50% (see Fig. 2). A positive PSE (rightward deviation of PSE) indicated that the individual made more left responses perceiving faces as looking to the left-sided space of observers (but the right-sided space of faces), which was defined as a leftward perception bias of gaze direction in the present study. On the contrary, a negative value indicated that the individual made more right responses, leading to a rightward perception bias of gaze direction.

SPSS 20 was used to analyze the data. Firstly, one-sample *t*-tests were conducted to compare respectively the means of PSE for four emotions with zero. Then, a one-way repeated measures analysis of variance (ANOVA) was conducted to investigate whether the judgment of gaze direction was modulated by emotion. Bonferroni correction was performed for all multiple comparisons.

### Results

The one-sample *t* tests showed that the PSE for fearful faces was significantly positive relative to zero, *t* (41) = 3.644, *p* = 0.001 (corrected *p* = 0.004), Cohen’s *d* = 0.562, indicating a left-sided bias in perception of gaze direction. The PSE for angry, happy, and neutral faces were not significantly different from zero (angry: *t* (41) = 0.433, *p* = 0.667 (1), Cohen’s *d* = 0.095; happy: *t* (41) = 0.521, *p* = 0.605 (1), Cohen’s *d* = 0.114; neutral: *t* (41) = 0.01, *p* = 0.992 (1), Cohen’s *d* = 0.002).

**Figure 3:**
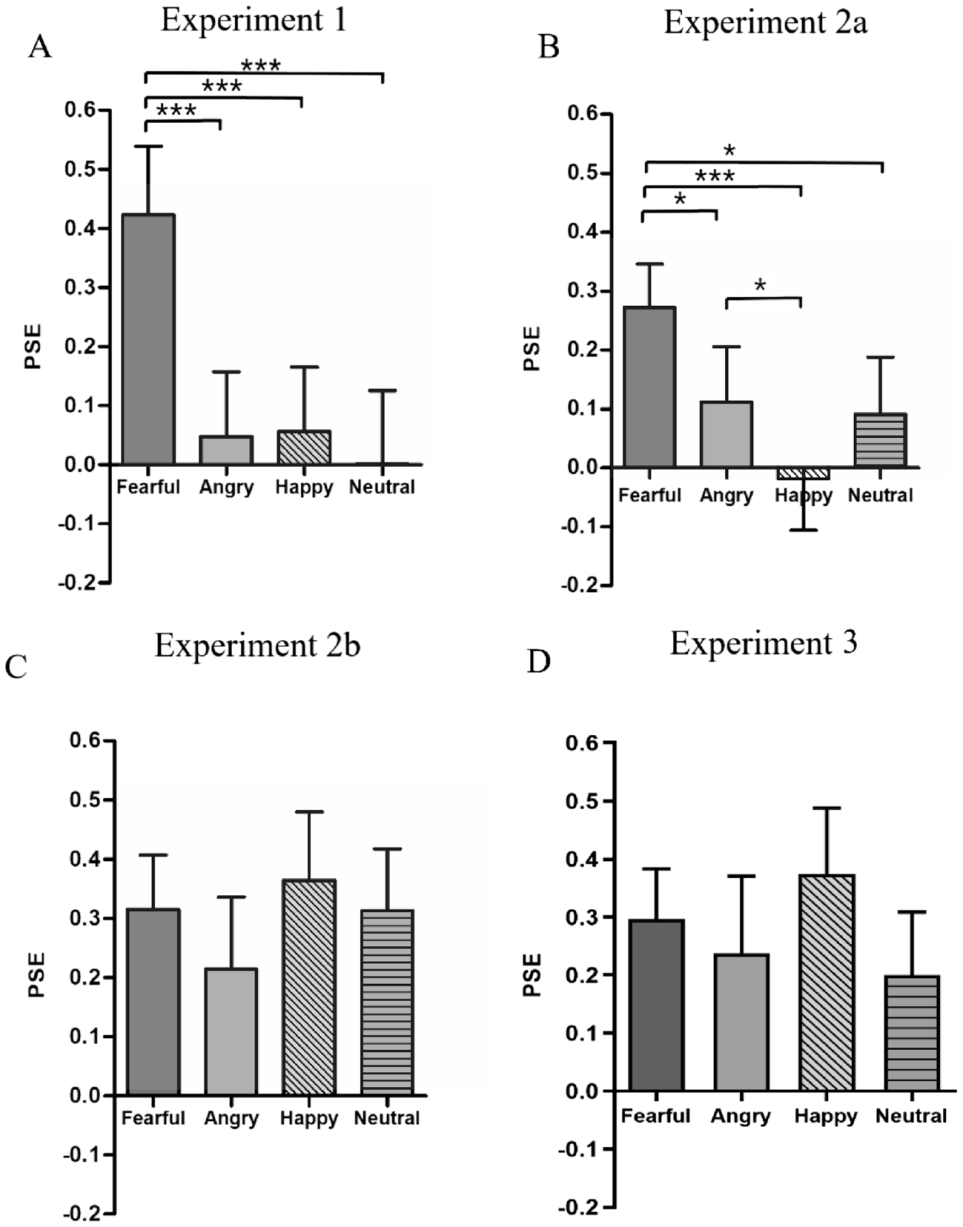
The means of the point of subjective equivalence (PSE) for four emotions in experiments 1 (panel **A**), 2a (panel **B**), 2b (panel **C**), and 3 (panel **D**). *p < 0.05; ***p < 0.001. Error bars represent SE.

The ANOVA showed that the main effect of emotion was significant, *F*(3,123) = 10.392, *p* < 0.001 ${\eta }_{p}^{2}$ = 0.202 (see Table 2). Pairwise comparisons with Bonferroni correction revealed that the PSE differences between fearful faces and all the other three emotions were significant (fearful vs. angry: *p* < 0.001, fearful vs. happy: *p* < 0.001, fearful vs. neutral: *p* < 0.001) while no significant differences were found between the other three emotions (angry vs. happy: *p* = 0.912, angry vs. neutral: *p* = 0.626, happy vs. neutral: *p* = 0.546) (see Fig. 3A).

**Table 2:** The point of subjective equivalence (*M* ± SD) of four facial expressions for experiments 1, 2a, 2b, and 3.

	Fearful	Angry	Happy	Neutral	*F*	*p*
Experiment 1	0.423 ± 0.116 ***	0.048 ± 0.11	0.057 ± 0.109	0.001 ± 0.124	10.392	< 0.001
Experiment 2a	0.272 ± 0.074 ***	0.112 ± 0.094	− 0.019 ± 0.087	0.091 ± 0.097	6.662	< 0.001
Experiment 2b	0.315 ± 0.092 **	0.214 ± 0.122 ^#^	0.365 ± 0.116 **	0.313 ± 0.105 **	1.479	0.228
Experiment 3	0.296 ± 0.087 ***	0.237 ± 0.133 ^#^	0.374 ± 0.113 ***	0.2 ± 0.108 ^#^	1.023	0.373

Significant differences between the means of point of subjective equivalence for each emotion and zero are indicated using asterisks (*p < 0.05, **p < 0.01, ***p < 0.001), and ^#^stands for marginally significance.

## Experiment 2a

In experiment 1, in order to control for the Simon effect, we required all participants to press “1” with the index finger for “looking left” and press “2” with the middle finger for “looking right” without counterbalancing the key press between participants. However, index finger may be better than middle finger in flexibility, which may make participants more inclined to use the index finger to make responses when perceiving an ambiguous gaze direction. If that is the case, the left-sided perception bias of gaze direction we achieved in experiment 1 might be confounded by the finger dexterity. To rule out this possibility, we designed experiment 2 with the corresponding responses counterbalanced between participants.

### Methods

#### Participants

Sixty-two participants from Liaoning Normal University of China were recruited. In this experiment, we counterbalanced the key-response settings across participants, which may weaken the experimental effect. In order to obtain the experimental effect comparable to that in experiment 1, we enlarged the sample size approximately to the largest sample size (i.e., 58) in previous studies^33^. Two of the participants were left-handed and another two muddled the responding keys for “looking left” and “looking right” during the test; therefore, four participants were excluded, leaving 58 participants (21 females, *M* ± SD = 21.1 ± 2.35 years) in the final analysis. All the participants had normal or corrected-to-normal vision and were right-handed according to their self-report. Signed informed consent was provided by each participant before the experiment. The study was conducted in accordance with the Declaration of Helsinki and was approved by the ethics committee of Liaoning Normal University (the approval number: lNNUIRB1720).

#### Materials

Same as experiment 1.

#### Procedure

The procedure was the same as experiment 1, except for the key press. All participants were asked to press “n” using the right index finger and press “j” using the right middle finger. However, the correspondence between keys and “left” and “right” responses was counterbalanced across participants.

#### Data analysis

Same as experiment 1.

### Results

The PSE for fearful faces was significantly positive relative to zero, *t*(57) = 3.701, *p* < 0.001 (0.002), Cohen’s *d* = 0.485, indicating a left-sided bias in perception of gaze direction. The PSE for angry, happy, and neutral faces were all not significant than zero (angry: *t* (57) = 1.196, *p* = 0.237 (0.946), Cohen’s *d* = 0.222; happy: *t* (57) = − 0.219, *p* = 0.827 (1), Cohen’s *d* = − 0.041; neutral: *t* (57) = 0.941, *p* = 0.351 (1), Cohen’s *d* = 0.175). The ANOVA showed that the main effect of emotion was significant, *F*(3,171) = 6.662, *p* < 0.001, ${\eta }_{p}^{2}$= 0.105 (Table 2). The PSE for fearful faces was significantly more positive than that in the other three emotions (fearful vs. angry: *p* = 0.023, fearful vs. happy: *p* < 0.001, fearful vs. neutral: *p* = 0.018), *p*s < 0.05, and the PSE for angry faces was more positive than that for happy faces, *p* = 0.036. Besides, the difference of PSE between happy and neutral face was marginally significant, *p* = 0.08, while such contrast between happy and neutral face was nonsignificant, *p* = 0.739 (Fig. 3B).

## Experiment 2b

In experiments 1 and 2a, we observed a significant left-sided bias in perception of gaze directions in fearful faces, excluding finger dexterity effects, and this bias was more positive than that for angry, happy, and neutral faces. However, it was not clear whether this advantage of fearful faces for the left-sided bias could be attributed to configural information, notably emotional information, or low-level featural information of faces. Face inversion is one of the most frequently used manipulations to separate configural or feature information processing of face^40^. The generally accepted explanation for the face inversion effect is that, when presented in the upright orientation, faces are processed mainly based on both configural and featural information, and differently inverted faces are processed mainly based on featural information. In other words, face inversion could hinder configural information processing. Configural information plays a prominent role in facial emotion processing^41^. Thus, face inversion could disrupt emotional information processing while retaining the features information processing. This is supported by Prkachin^42^ reporting that the sensitivity to inverted expressions was diminished for all six emotions (happiness, sadness, anger, disgust, surprise, and fear) when subjects were asked to detect and label facial expressions of emotion. Given the validity of the inversion approach, our study directly applied the approach to eyes. We presented face images upside down to disrupt the processing of emotional information while retaining the processing of facial features^43,44^ to address above question. If the advantage of the left-sided bias in the fearful condition was due to the emotional information expressed by fearful faces, we would not observe significant PSE differences between fear and other three emotion conditions following the presentation of inverted faces.

### Methods

#### Materials

The faces used experiment 2b were the same with those in experiments 1 and 2a, except that the faces were rotated 180°, as shown in Fig. 1B.

#### Participants and procedure

Same as experiment 2a. Participants were instructed to judge whether inverted faces were looking to observers’ left-sided or right-sided space.

#### Data analysis

Same as experiments 1 and 2a.

### Results

The PSE of inverted fearful, *t*(57) = 3.431, *p* = 0.001 (0.004), Cohen’s *d* = 0.45, happy, *t*(57) = 3.156, *p* = 0.003 (0.01), Cohen’s *d* = 0.415, and neutral faces, *t*(57) = 2.985, *p* = 0.004 (0.017), Cohen’s *d* = 0.392, were significantly more positive than zero, and the PSE of inverted angry face was marginally positive as compared to zero, *t*(57) = 1.752, *p* = 0.085 (0.341), Cohen’s *d* = 0.23 (see Table 2). The ANOVA showed that the main effect of emotion was not significant, *F*(3,171) = 1.479, *p* = 0.228 (see Fig. 3C), indicating that inverting the faces eliminated the bias advantage for fearful faces.

## Experiment 3

In experiment 2b, we found left-sided biases in perception of gaze directions in all types of faces which were inverted to exclude the impact of emotional information. However, inversion is known to selectively hinder configural processing, not exclude all other facial features influences. Besides, according to Bombari et al*.*^41^, the recognition of different emotions relies on different types of facial information; for example, the mouth is important for the detection of happiness and fear. Thus, face inversion could not eliminate the effect of face context on the experimental results. To examine whether the leftward perception biases were contributed by gaze per se, we used inverted eyes alone to eliminate all other facial features’ influence in experiment 3 and expected to observe significant PSE differences between each of the four conditions and the zero baseline.

### Methods

#### Materials

Eyes of the inverted faces in experiment 2b were used, with the other parts of faces wiped out (see Fig. 1B).

#### Participants and procedure

Sixty participants from Liaoning Normal University of China were recruited. The sample size was determined in the same way as that in Experiment 2. Two of them muddled the responding keys for “looking left” and “looking right” during the experiment and the performance of another two participants fell out of three SDs of normal distribution; therefore, they were excluded, leaving 56 participants (42 females, M ± SD = 21.2 ± 1.96 years) in the final analysis. Participants had normal or corrected-to-normal vision and were all right-handed according to their self-report. Signed informed consent was provided by each participant before the experiment. The study was conducted in accordance with the Declaration of Helsinki and was approved by the ethics committee of Liaoning Normal University (the approval number: lNNUIRB2020). Participants were instructed to judge whether inverted eyes were looking to the left-sided or right-sided space of observers.

#### Data analysis

Same as experiments 1 and 2.

### Results

The PSE of inverted fearful eyes, *t*(55) = 3.393, *p* = 0.001 (0.005), Cohen’s *d* = 0.642, and happy eyes, *t*(55) = 3.307, *p* = 0.002 (0.007), Cohen’s *d* = 0.624, were significantly more positive than zero, and the PSE of inverted angry eyes, *t*(55) = 1.779, *p* = 0.081 (0.323), Cohen’s *d* = 0.336, and neutral eyes, *t*(55) = 1.852, *p* = 0.069 (0.277), Cohen’s *d* = 0.35, was marginally positive as compared to zero (Table 2). The ANOVA showed that the main effect of emotion was not significant, *F*(3,165) = 1.023, *p* = 0.371 (see Fig. 3D).

## Discussion

In this study, three behavioral experiments were conducted to investigate whether there would be a left-sided bias in perception of gaze direction and whether this bias would be modulated by face emotion. The results of experiments 1 and 2a showed a left-sided bias in the processing of others’ gaze direction in fearful faces, suggesting that individuals are likely to judge others expressing fear as looking at their (observers’) left side, whereas this bias did not occur in angry, happy, or neutral faces. These findings are in line with previous research showing emotion-dependent perception of gaze direction^12,14,31,37^. For example, Adams and Franklin^12^ found that averted relative to direct gaze was processed more quickly and accurately when coupled with fear and direct relative to averted gaze with anger. Using the cone of direct gaze (CoDG), the range of eye deviations that observers judge as being directed toward them, Shi et al*.*^31^ showed that fearful faces were less judged as having direct gaze than angry and neutral faces. In a forced-choice yes–no task, Lobmaier et al*.*^14^ found that happy and angry faces were more often judged as looking at the observers than were fearful and neutral expressions.

The fear-specific leftward bias of gaze direction perception observed here may be associated with survival mechanisms in threatening situations including right hemisphere dominance in processing novel and unpredictable threatening stimuli^45^. Martín et al*.*^25^ found that lizards had a tendency to run to their right-hand side when they escaped from a predator and suggested that the rightward escape bias could be induced by a preference to keep the potential dangers in the left-sided space. Chicks prefer to use their left eye to monitor the gaze of dummy masks^46^, which bases on the left-hemispace attention allocation bias of birds that has been also demonstrated in other paradigms^47,48^. Pigeons tend to fly to the right of their favourite flight-partners, monitoring them with their left eye and keeping them in their left space to avoid potential environmental harm happening to their lovers when homing in flocks^49^. Similarly, in sea mammals, a bias to monitor social signals from conspecifics with the left eye has been observed during mother–offspring interactions^50^. Our findings consist with such behavioral asymmetries in animal social recognition^51^. The preference to perceive fearful faces as looking left here may reflect observers’ tendency to believe that the possible danger implied by threatened faces is in the left-sided space. By closely monitoring the left space, individuals can react to the threats quickly and reduce the consequences and losses.

Another possible explanation is that the leftward bias for fearful faces may be related to valence-specific or motivation-dependent hemispheric asymmetry. Fear is a negative emotion and associated with avoidance motivation. Previous studies have shown that the right hemisphere plays a dominant role in processing negative emotions^28,52^ and avoidance motivation^53^. This right hemisphere dominance may affect perception, causing individuals to make more left-sided judgments of gaze direction for fearful faces. However, this explanation doesn’t explain why a comparable leftward bias of gaze direction perception wasn’t observed in angry faces which express negative emotion. Moreover, if the right hemisphere dominance for avoidance motivation contributes to the leftward bias for fearful faces, then neutral faces should display a moderate tendency between fear and happy expressions. In fact, we found no significant difference in PSE between neutral and happy faces. Therefore, we contend that the underlying processes of the fear-specific leftward bias of gaze direction perception might be rather complex and needs further study in the future.

It is noteworthy that gaze direction perception in angry faces differed from that in fearful faces although one might expect similar leftward preferences in angry and fearful faces given their relations to threat, avoidance behavior (from the observer’s point of view)^54^, and the dominance of the right hemisphere in processing negative emotions^28,52^. A few studies showed that anger and fear could trigger some analogous reactions of individuals, such as stronger attention guiding^55^ and better memory^56,57^ of angry and fearful faces as compared to neutral conditions. However, angry and fearful faces indeed convey different information of threat, with anger for immediate menace but fear for potential risk in the environment^58^. The neural substrates underlying the perception of anger and fear have been strongly suggested to be dissociable^59^. Therefore, the different perception of gaze direction in angry and fearful faces (in experiment 1 and 2a) is consistent with both our predictions and previous findings showing differentiated perception of gaze direction in angry and fearful faces^12,14,31^.

Using inverted stimuli, we found leftward gaze perception biases in experiments 2b and 3 regardless of expressions. This indicates that, following elimination of emotional information by inversion, the emotion modulation of gaze direction judgments disappeared, and the leftward gaze perception bias was caused by gaze itself rather than other information in face. Also, the presence of leftward biases in all conditions suggests that humans may generally tend to mistake others’ gaze as directed to the left-sided space of observers. This is in line with part of the findings in Calder et al*.*^60^ suggesting a discrepancy between actual and perceived gaze direction, with more accurate categorization of left relative to right gaze and an increased tendency to categorize direct gaze as left than right, although the study primarily aimed at functional accounts for visual perception of gaze in humans instead of gaze perception asymmetry. The universal leftward gaze perception bias may be related with the right hemispheric dominance in gaze processing^61,62^. For example, Okada et al*.*^62^ tested such superiority using a target localization task, with preceding non-predicative gaze cues presented to each visual field. Their results showed that the cuing effect was significantly larger for the left than right visual field presentation, supporting a right hemispheric dominance in the gaze processing that mediates attentional shift. In our experiments, participants were required to judge the direction of face stimuli and such judgments included similar gaze-triggered attention shifts. Thus, the right hemisphere dominance of gaze processing may induce a general leftward gaze direction perception bias, considering that right hemisphere is responsible for the information processing in the left visual filed.

The universal leftward bias of gaze direction perception, indexed by the rightward deviation of PSE, appears inconsistent with the “pseudoneglect” phenomenon, a subtle bias of visual attention favoring left space, which results in a leftward center deviation bias when bisecting non-social stimuli, like lines and numbers^20,21^. The inconsistency between the rightward deviation of the theoretical center point (i.e., PSE) of gaze perception and the leftward deviation of the subjective center point of non-social stimuli may be related to the different neural mechanisms of attention processing in gaze and non-social physical stimuli. Vuilleumier^63^ found that parietal damage causes spatial neglect and impairs the representation of location on the contralesional side. However, perceived gaze in faces could still trigger automatic shifts of attention in the contralesional direction while symbolic arrow cues had only minimal or no effect, suggesting the existence of specific and anatomically distinct attentional mechanisms of gaze. Asking participants to identify the direction of laterally presented stimuli, Marotta et al*.*^64^ found opposite behavioral and electrophysiological effects for eye-gaze and non-social arrow stimuli. These findings suggest that attention triggered by eye-gaze may represent a process different from that triggered by non-social stimuli, thus producing inconsistency in perception asymmetry for gaze direction here and line bisection. Nevertheless, it is worth noting that the asymmetries in gaze perception and line bisection may not be necessarily inconsistent, considering the possibility that face observers focus more on their left hemispace and therefore assume a face directed at them also looks at this attended hemispace.

Given the universal leftward perception bias of gaze direction in experiments 2b and 3, we assume that the absence of gaze perception bias in angry, happy, and neutral faces in experiments 1 and 2a may be due to a leftward deviation of PSE induced by emotional information in these faces. Such emotion-dependent leftward deviation of PSE offsets the general gaze-induced rightward deviation of PSE, leading to an unbiased PSE. In other words, participants are more prone to judge angry, happy, and neutral faces as looking to the right-sided space of themselves, which is compatible with other rightward behavioral biases. For example, Yuan et al*.*^65^ examined turning bias in navigating a virtual Morris Water Maze and showed that all participants veered to the right. Nicholls et al*.*^66^ identified a rightward asymmetry in collisions in operating a wheelchair and this right-side bias has been also supported by animal studies^67^. Two previous studies analyzed behavioral biases during penalty shootouts and found that when the goalkeeper's team was behind the goalkeeper dived to the right more often than to the left^68,69^. The rightward behavioral biases may share an evolutionary and adaptive mechanism with our rightward gaze perception bias induced by angry, happy, and neutral faces. One possible reason is that about 90% humans are right-handed^4^ and there the biases may allow humans to use their dominant hand to achieve efficient reaction and thus improve the chances to survive and gain more benefits.

Besides, as the eyes widely open for fearful faces, the size contrast between iris and sclera was larger for fearful faces than for faces with other expressions. However, such contrast cannot explain the results of experiment 2b and 3 that no significant differences were found between fearful face and any other three emotions, considering that face and eyes inversion does not change the physical contrast between iris and sclera. Thus, we contend that it was emotional information, not the low-level featural information of faces, that produced such differences between fearful face and others.

As for fearful faces, one might expect that the leftward bias in experiments 1 and 2a would be magnified by the universal leftward bias in experiments 2b and 3. This did not happen possibly due to a ceiling effect in the bias measurement. Another possible explanation is that there was no bias induced by fearful expression and the fear-specific leftward bias observed in experiments 1 and 2a was indeed the gaze-induced general leftward bias. Future studies could use different designs to test the two hypotheses.

Overall, the current research provides evidence that there exists a fear-specific left-ward bias in processing eye gaze direction, which may be a sign of an evolutionary cognitive bias associated with threats and supports the lateralization of the human brain in processing social information. A limitation in our study is that we did not balance the order of the tasks using upright and inverted stimuli in experiment 2 and therefore failed to exclude fatigue or learning effects. Moreover, we presented virtual faces of Caucasian rather to Asian individuals, possibly introducing potential confounds due to other-race effects. We only selected 3 male and 3 female faces, which may pose obstacles for us to generalize our conclusions. Future studies should use a more delicate design to rule out the confounding effects.

## Supplementary Information

Supplementary Tables.

## Data Availability

The datasets generated during and/or analysed during the current study are available from the corresponding author (huzhonghua2000@163.com, Zhonghua Hu) on reasonable request.
