# Supplementary material for: Fear-specific leftward bias in gaze direction judgment
Source: Sci Rep. 2021 Sep 2;11:17574. doi: 10.1038/s41598-021-97039-3 (PMC8413379; doi:10.1038/s41598-021-97039-3)
Supplement: Supplementary file 1 — Supplementary Tables. [file 41598_2021_97039_MOESM1_ESM.docx]

**Table 1** Probabilities (*M* ± SD) of “looking at left” responses for four emotional faces in Experiment 1.

| Emotion | | Gaze direction | | | | | | | | | |
| --- | --- | --- | --- | --- | --- | --- | --- | --- | --- | --- | --- |
|  |  | Left - 5 | Left - 4 | Left - 3 | Left - 2 | Left - 1 | Right - 1 | Right - 2 | Right - 3 | Right - 4 | Right - 5 |
| Fearful | 0.99 ± 0.02 | | 0.98 ± 0.07 | 0.98 ± 0.05 | 0.94 ± 0.08 | 0.85 ± 0.18 | 0.35 ± 0.19 | 0.14 ± 0.16 | 0.06 ± 0.08 | 0.03 ± 0.06 | 0.02 ± 0.06 |
| Angry | 0.99 ± 0.03 | | 0.97 ± 0.05 | 0.96 ± 0.07 | 0.89 ± 0.13 | 0.76 ± 0.15 | 0.29 ± 0.17 | 0.14 ± 0.12 | 0.06 ± 0.11 | 0.03 ± 0.06 | 0.01 ± 0.08 |
| Happy | 0.99 ± 0.06 | | 0.99 ± 0.05 | 0.97 ± 0.1 | 0.92 ± 0.1 | 0.78 ± 0.16 | 0.29 ± 0.17 | 0.13 ± 0.12 | 0.05 ± 0.1 | 0.03 ± 0.06 | 0.01 ± 0.05 |
| Neutral | 1 ± 0.03 | | 0.97 ± 0.05 | 0.96 ± 0.09 | 0.91 ± 0.12 | 0.74 ± 0.18 | 0.3 ± 0.19 | 0.11 ± 0.13 | 0.05 ± 0.09 | 0.02 ± 0.04 | 0.01 ± 0.03 |

**Table 2** Probabilities (*M* ± SD) of “looking at left” responses for four emotional faces in Experiment 2.

| Emotion | | Gaze direction | | | | | | | | | |
| --- | --- | --- | --- | --- | --- | --- | --- | --- | --- | --- | --- |
|  |  | Left - 5 | Left - 4 | Left - 3 | Left - 2 | Left - 1 | Right - 1 | Right - 2 | Right - 3 | Right - 4 | Right - 5 |
| Fearful | 0.98 ± 0.04 | | 0.98 ± 0.05 | 0.98 ± 0.05 | 0.94 ± 0.09 | 0.82 ± 0.14 | 0.28 ± 0.15 | 0.09 ± 0.1 | 0.05 ± 0.07 | 0.02 ± 0.04 | 0.01 ± 0.03 |
| Angry | 0.98 ± 0.04 | | 0.97 ± 0.06 | 0.93 ± 0.1 | 0.87 ± 0.13 | 0.77 ± 0.15 | 0.29 ± 0.17 | 0.15 ± 0.12 | 0.04 ± 0.07 | 0.02 ± 0.04 | 0.02 ± 0.05 |
| Happy | 0.98 ± 0.04 | | 0.98 ± 0.04 | 0.96 ± 0.06 | 0.9 ± 0.11 | 0.76 ± 0.17 | 0.24 ± 0.15 | 0.09 ± 0.1 | 0.04 ± 0.06 | 0.02 ± 0.04 | 0.01 ± 0.03 |
| Neutral | 0.98 ± 0.04 | | 0.98 ± 0.05 | 0.96 ± 0.07 | 0.92 ± 0.11 | 0.78 ± 0.16 | 0.27 ± 0.18 | 0.1 ± 0.11 | 0.05 ± 0.08 | 0.02 ± 0.05 | 0.02 ± 0.03 |

**Table 3** Probabilities (*M* ± SD) of “looking at left” responses for four emotional faces in Experiment 2b.

| Emotion | | Gaze direction | | | | | | | | | |
| --- | --- | --- | --- | --- | --- | --- | --- | --- | --- | --- | --- |
|  |  | Left - 5 | Left - 4 | Left - 3 | Left - 2 | Left - 1 | Right - 1 | Right - 2 | Right - 3 | Right - 4 | Right - 5 |
| Fearful | 0.98 ± 0.05 | | 0.98 ± 0.04 | 0.97 ± 0.05 | 0.91 ± 0.11 | 0.79 ± 0.17 | 0.31 ± 0.16 | 0.16 ± 0.15 | 0.07 ± 0.1 | 0.03 ± 0.06 | 0.02±0.04 |
| Angry | 0.97 ± 0.06 | | 0.95 ± 0.08 | 0.92 ± 0.1 | 0.86 ± 0.13 | 0.73 ± 0.18 | 0.33 ± 0.17 | 0.19 ± 0.15 | 0.1 ± 0.12 | 0.07 ± 0.1 | 0.04±0.06 |
| Happy | 0.98 ± 0.04 | | 0.97 ± 0.06 | 0.95 ± 0.08 | 0.89 ± 0.11 | 0.77 ± 0.16 | 0.34 ± 0.2 | 0.18 ± 0.15 | 0.1 ± 0.13 | 0.05 ± 0.08 | 0.03±0.06 |
| Neutral | 0.98 ± 0.04 | | 0.97 ± 0.06 | 0.94 ± 0.1 | 0.87 ± 0.13 | 0.79 ± 0.17 | 0.34 ± 0.18 | 0.16 ± 0.14 | 0.08 ± 0.1 | 0.03 ± 0.05 | 0.03±0.05 |

**Table 4** Probabilities (*M* ± SD) of “looking at left” responses for four emotional faces in Experiment 3.

| Emotion | | Gaze direction | | | | | | | | | |
| --- | --- | --- | --- | --- | --- | --- | --- | --- | --- | --- | --- |
|  |  | Left - 5 | Left - 4 | Left - 3 | Left - 2 | Left - 1 | Right - 1 | Right - 2 | Right - 3 | Right - 4 | Right - 5 |
| Fearful | 0.96 ± 0.13 | | 0.96 ± 0.12 | 0.94 ± 0.11 | 0.89 ± 0.14 | 0.76 ± 0.17 | 0.34 ± 0.18 | 0.17 ± 0.17 | 0.1 ± 0.15 | 0.05 ± 0.11 | 0.05 ± 0.12 |
| Angry | 0.95 ± 0.11 | | 0.93 ± 0.14 | 0.89 ± 0.15 | 0.83 ± 0.16 | 0.74 ± 0.14 | 0.36 ± 0.18 | 0.23 ± 0.17 | 0.14 ± 0.15 | 0.09 ± 0.15 | 0.06 ± 0.13 |
| Happy | 0.95 ± 0.12 | | 0.93 ± 0.14 | 0.92 ± 0.13 | 0.89 ± 0.14 | 0.77 ± 0.15 | 0.36 ± 0.19 | 0.21 ± 0.15 | 0.14 ± 0.16 | 0.09 ± 0.14 | 0.07 ± 0.13 |
| Neutral | 0.96 ± 0.11 | | 0.95 ± 0.13 | 0.92 ± 0.12 | 0.85 ± 0.14 | 0.73 ± 0.17 | 0.35 ± 0.2 | 0.18 ± 0.16 | 0.11 ± 0.15 | 0.06 ± 0.13 | 0.05 ± 0.12 |
